# Occurrence of Slow Flow/No-Reflow in Primary Percutaneous Coronary Intervention: Predictors, Management, and Outcomes

**DOI:** 10.7759/cureus.99180

**Published:** 2025-12-14

**Authors:** Shama Ayaz, Hidayat Ullah, Fahad R Khan, Mohammad Waleed, Rafi Ullah Jan, Imran Ali, Abid Ullah

**Affiliations:** 1 Interventional Cardiology, Peshawar Institute of Cardiology, Peshawar, PAK; 2 Interventional Cardiology, Manchester Royal Infirmary, Manchester, GBR

**Keywords:** in-hospital outcomes, no-reflow, primary pci, slow flow, st-segment elevation myocardial infarction, thrombus burden, timi flow

## Abstract

Background

Slow flow/no-reflow (SF/NR) can undermine effective reperfusion during primary percutaneous coronary intervention (PCI) for ST-segment elevation myocardial infarction (STEMI). Identifying patients at risk before device escalation may enable protocolized microvascular-protection strategies within STEMI systems of care.

Objective

This study aimed to determine the prevalence of SF/NR in STEMI treated with primary PCI, identify pre-PCI predictors, and assess short-term in-hospital outcomes associated with SF/NR.

Methods

In a prospective cohort at a high-volume tertiary center, we enrolled 226 consecutive STEMI patients treated between January 1, 2024, and December 31, 2024 (complete-case analysis). SF/NR was defined as final thrombolysis in myocardial infarction (TIMI) flow <3 in the absence of mechanical obstruction. Multivariable logistic regression estimated independent predictors (adjusted odds ratios (aORs) with 95% CIs). Discrimination was assessed using the area under the receiver operating characteristic curve (AUC) with bootstrap optimism correction. In-hospital outcomes included final TIMI 3 flow, ventricular arrhythmia, hemodynamic instability, heart failure, and death.

Results

SF/NR occurred in 65/226 patients (28.8%). Independent predictors were diabetes mellitus (aOR, 2.05; 95% CI, 1.07-3.91), TIMI thrombus grade 5 (aOR, 3.02; 95% CI, 1.55-5.87), and pre-PCI TIMI 0 flow (aOR, 3.85; 95% CI, 1.53-9.67); symptom-to-balloon time >6 h was not an independent predictor. Model discrimination was fair (AUC, 0.74; 95% CI, 0.67-0.81; optimism-corrected AUC, 0.73). Compared with patients without SF/NR, those with SF/NR had lower final TIMI 3 flow (43.1% vs 82.0%; difference, −38.9 percentage points; 95% CI, −51.0 to −26.8) and higher rates of ventricular arrhythmia (18.5% vs 5.6%; P = 0.004), hemodynamic instability (24.6% vs 8.6%; P = 0.002), heart failure (21.5% vs 9.3%; P = 0.01), and in-hospital mortality (7.7% vs 1.9%; P = 0.04).

Conclusions

In contemporary primary PCI for STEMI, SF/NR was common and associated more strongly with thrombus burden and absent antegrade flow, along with diabetes, than with delays >6 hours. A simple pre-PCI triad (diabetes, TIMI thrombus grade 5, and TIMI 0 flow) may help flag higher-risk cases and prompt early microvascular-protection measures; external validation is warranted prior to routine adoption.

## Introduction

Coronary artery disease remains a leading cause of death worldwide, and ST-segment elevation myocardial infarction (STEMI) is its most time-critical presentation. Primary percutaneous coronary intervention (PCI) reliably re-establishes epicardial patency and reduces mortality; however, outcomes may be undermined by the slow-flow/no-reflow (SF/NR) phenomenon, angiographic thrombolysis in myocardial infarction (TIMI) flow <3 with inadequate microvascular perfusion despite relief of epicardial obstruction [1].

Pathophysiologically, SF/NR reflects downstream microvascular obstruction rather than residual epicardial stenosis. Contributory mechanisms include distal atherothrombotic embolization, endothelial swelling and spasm, platelet-leukocyte plugging, and ischemia-reperfusion injury, each of which impairs tissue-level perfusion after otherwise successful epicardial recanalization [2].

The clinical relevance is substantial. Across contemporary STEMI cohorts, SF/NR is consistently associated with worse in-hospital and longer-term outcomes, including higher mortality and major adverse cardiovascular events, than cases achieving normal reperfusion after primary PCI [3,4]. Accordingly, early recognition and prevention are emphasized in STEMI systems of care.

Therapeutic options used at the time of SF/NR include intracoronary vasodilators (adenosine, nitrates, and non-dihydropyridine calcium-channel blockers), selective adrenergic rescue (e.g., epinephrine in refractory cases), and intensive antiplatelet regimens (e.g., glycoprotein IIb/IIIa inhibitors). Comparative head-to-head evidence remains limited, and no single regimen reliably reverses established no-reflow; guideline-concordant practice therefore favors prevention, prompt identification, and pragmatic, stepwise treatment algorithms [5-7].

Signals from small studies suggest diltiazem may outperform nitrates for improving flow in slow-flow contexts, but estimates are imprecise and not specific to STEMI no-reflow; thus, any agent preference should be cautious and individualized [8]. Pharmacotherapy can be delivered proximally via the guide or distally via a microcatheter to enhance local bioavailability; available reports are supportive but methodologically heterogeneous, and no definitive standard has emerged [9].

Against this background, we aimed to determine the prevalence of SF/NR in STEMI patients undergoing primary PCI, identify pre-PCI predictors, and evaluate short-term in-hospital outcomes of SF/NR in a contemporary, high-volume tertiary care setting.

## Materials and methods

Study design, setting, and oversight

We conducted a prospective observational cohort at the Peshawar Institute of Cardiology, Khyber Pakhtunkhwa, Pakistan, a high-volume tertiary cardiac center and contributor to the national PCI registry. The primary objective was to estimate the frequency and correlates of SF/NR after primary PCI for STEMI. The a priori sample size for a single proportion assumed an expected SF/NR prevalence of 18%, a 95% confidence level, and absolute precision of 5%, yielding a target of 226 participants using the standard formula for prevalence studies [10]. Consecutive eligible patients presenting between January 1, 2024, and December 31, 2024 (12 months) were enrolled. The protocol was approved by the institutional review board of the institution (approval number: IRC/24/75). Written informed consent was obtained from all participants. The study's conduct adhered to the Declaration of Helsinki, and reporting followed Strengthening the Reporting of Observational Studies in Epidemiology (STROBE) guidance [11,12].

Participants

Adults with STEMI triaged directly to the catheterization laboratory for primary PCI were screened. Inclusion required treatment of the culprit lesion using a drug-eluting stent or drug-eluting balloon. Exclusions were receipt of thrombolysis without subsequent mechanical reperfusion, balloon-only primary PCI, or prior coronary artery bypass grafting with planned intervention of a saphenous vein graft. Eligible patients were enrolled consecutively and followed through hospital discharge; analyses used the complete-case cohort.

Periprocedural care and angiography

At presentation, demographics, comorbidities, total ischemia time (symptom onset to first balloon inflation), and focused clinical history were recorded. Unless contraindicated, patients received aspirin (300 mg) and clopidogrel (600 mg) orally plus weight-based unfractionated heparin (100 IU/kg intravenously) prior to catheterization, in line with the institutional STEMI protocol. Use of glycoprotein IIb/IIIa inhibitors and intracoronary vasodilators (e.g., nitrates, adenosine, or calcium-channel blockers) was not mandated but was available and applied at the discretion of the treating interventional cardiologist. Vascular access (radial or femoral) and device strategy (e.g., stent implantation, aspiration thrombectomy, adjunctive devices) were left to operator judgment. Aspiration thrombectomy, post-dilatation, and other adjunctive devices were likewise employed according to operator preference rather than a prespecified stepwise protocol. All procedures were performed by consultant interventional cardiologists or senior fellows under direct supervision.

The culprit artery was identified angiographically. Epicardial antegrade flow before and after intervention was graded using the TIMI flow scale (0-3) [13]. Thrombus burden was quantified with the TIMI thrombus scale (grade 0, no visible thrombus; grade 5, complete occlusion) [14]. All angiograms were reviewed on-site by two experienced interventional cardiologists; TIMI flow and thrombus grades were assigned by consensus, with any initial discrepancies resolved on joint review. SF/NR was defined as post-stent TIMI 2 (slow flow) or TIMI 0-1 (no-reflow) at the index lesion in the absence of dissection, persistent/new thrombus, vasospasm, or high-grade residual stenosis. Intracoronary medications were administered for SF/NR, and the final angiographic result was recorded.

In-hospital monitoring and outcomes

Patients were observed for electrical events (tachyarrhythmias, bradyarrhythmias), hemodynamic instability (cardiogenic shock, heart failure), and renal injury. Acute kidney injury was defined per the Kidney Disease: Improving Global Outcomes (KDIGO) criteria [15]. Transthoracic echocardiography was obtained when clinically indicated. The in-hospital outcome set comprised final TIMI 3 flow, ventricular arrhythmia, hemodynamic instability, heart failure, and all-cause mortality.

Statistical analysis

Analyses were performed with IBM SPSS Statistics software, version 27.0 (IBM Corp., Armonk, NY) [16]. Continuous variables are summarized as medians with interquartile ranges (IQRs) and compared using the Mann-Whitney U test; categorical variables are presented as counts (percentages) and compared using Fisher's exact tests. Univariable relative risks with 95% confidence intervals were estimated for candidate predictors of SF/NR. Variables meeting a screening threshold (P < .10) or judged clinically relevant were entered into a multivariable logistic regression model. Model discrimination was assessed by the area under the receiver operating characteristic curve (AUC) [17], with bootstrap optimism correction; calibration was evaluated with the Hosmer-Lemeshow goodness-of-fit test [18]. Multicollinearity was assessed via variance inflation factors (VIFs), targeting VIF < 2 [19], and we maintained an events-per-variable ratio ≥ 10:1 [20]. Two-sided tests used α = 0.05. To address potential overfitting, we additionally reported an optimism-corrected AUC estimated via bootstrap resampling [21].

## Results

Study population

Among 226 consecutive STEMI patients treated with primary PCI between January 1, 2024, and December 31, 2024, no cases were excluded for missing data; analyses were conducted on the complete-case cohort.

Baseline clinical, angiographic, and procedural characteristics

The median age was 59.5 (range: 52.0-67.0) years, and 172 patients of 226 (76.1%) were men. Hypertension was present in 133 (58.8%), diabetes mellitus in 86 (38.1%), and current smoking in 23 (10.2%) patients. A family history of coronary artery disease was reported by 45 (19.9%) patients. Ischemic time was prolonged (symptom-to-balloon: 7.44 hours (5.37-10.30 hours)). Anterior-wall infarction occurred in 113 (50.0%) and inferior-wall infarction in 103 (45.5%) patients. Angiography identified the left anterior descending artery as the culprit in 114 (50.4%); single-, double-, and triple-vessel disease occurred in 101 (44.7%), 78 (34.5%), and 46 (20.4%) patients, respectively. Pre-procedural TIMI 0 flow was observed in 149 (65.9%) patients, and TIMI thrombus grade 5 in 149 (65.9%) patients. Final TIMI 3 flow was achieved in 160 (70.8%) patients. In-hospital complications occurred in 36 (15.9%), most commonly cardiogenic shock (15; 6.6%) and tachyarrhythmias (12; 5.3%); in-hospital mortality was 13 (5.8%) patients, as shown in Table 1.

**Table 1:** Baseline clinical, angiographic, and procedural characteristics (N=226) Values are n (%) or median (IQR). This table summarizes baseline clinical, angiographic, and procedural characteristics for patients with STEMI undergoing primary PCI. Continuous variables are reported as median (IQR; non-normal distributions); categorical variables as n (%). No between-group hypothesis testing is presented for this descriptive table. Analyses were performed on the complete-case cohort (N = 226); no missing data were imputed. Abbreviations: CAD: coronary artery disease; AWMI: anterior wall myocardial infarction; IWMI: inferior wall myocardial infarction; LAD: left anterior descending artery; TIMI: thrombolysis in myocardial infarction; IQR: interquartile range.

Category	Variable	Value
Demographics	Age, years	59.5 (52.0–67.0)
	Male sex	172 (76.1%)
Risk factors	Hypertension	133 (58.8%)
	Diabetes mellitus	86 (38.1%)
	Smoking	23 (10.2%)
	Family history of CAD	45 (19.9%)
Clinical presentation	AWMI	113 (50.0%)
	IWMI	103 (45.5%)
Angiography	Single-vessel CAD	101 (44.7%)
	Double-vessel CAD	78 (34.5%)
	Triple-vessel CAD	46 (20.4%)
	Culprit vessel: LAD	114 (50.4%)
	Pre-procedural TIMI 0 flow	149 (65.9%)
	TIMI thrombus grade 5	149 (65.9%)
Procedural outcomes	Final TIMI 3 flow	160 (70.8%)
	In-hospital complications	36 (15.9%)
	Cardiogenic shock	15 (6.6%)
	Tachyarrhythmias	12 (5.3%)
	In-hospital mortality	13 (5.8%)

Incidence and management of SF/NR

SF/NR occurred in 65/226 (28.8%). TIMI grading was documented contemporaneously by two independent interventional cardiologists. Management was predominantly guide-catheter-delivered pharmacotherapy. Among SF/NR cases, intracoronary nitrates (27.9%) and tirofiban (27.4%) were most frequently administered, followed by adrenaline (12.8%) and adenosine (3.1%). No distal delivery systems or standardized preemptive vasodilator protocols were in place; aspiration thrombectomy use was not systematically captured. Despite treatment, optimal epicardial recovery remained uncommon in SF/NR, with a −38.9 percentage-point absolute difference in final TIMI 3 flow versus no SF/NR (43.1% vs 82.0%; P < 0.001), indicating limited reversibility once established. Clinical and angiographic characteristics stratified by the presence or absence of SF/NR are summarized in Table 2.

**Table 2:** Key clinical and angiographic variables by SF/NR status An asterisk indicates statistical significance (P < 0.05). This table compares key clinical and angiographic variables by SF/NR status after primary PCI. Continuous variables are reported as median (IQR) and compared with the Mann–Whitney U test; categorical variables are n (%) and compared with Fisher’s exact test. Effect sizes are presented as RRs with 95% CIs to convey the magnitude of association. Interpretation: prioritize effect sizes and CIs for clinical relevance; P-values are descriptive. Available case analysis with cell-level denominators shows if any category has missingness. Abbreviations: SF/NR: slow flow/no-reflow; RR: risk ratio; CI: confidence interval; TIMI: thrombolysis in myocardial infarction; IQR: interquartile range.

Category	Variable	No SF/NR (n = 161)	SF/NR (n = 65)	P-value	Effect
Clinical	Ischemia time, hours	7.38 (5.45–10.30)	8.08 (5.27–9.54)	0.015*	—
	Diabetes mellitus	53 (32.9%)	33 (50.8%)	0.019*	RR 1.54 (95% CI, 1.06–2.23)
Angiographic	TIMI thrombus grade 4	4 (2.5%)	8 (12.3%)	0.018*	RR 4.90 (95% CI, 1.50–15.50)
	Final TIMI 3 flow	132 (82.0%)	28 (43.1%)	<0.001*	RR 0.52 (95% CI, 0.39–0.69)
Outcomes	In-hospital mortality	6 (3.7%)	7 (10.8%)	0.045*	RR 2.90 (95% CI, 1.10–7.50)

**Figure 1:**
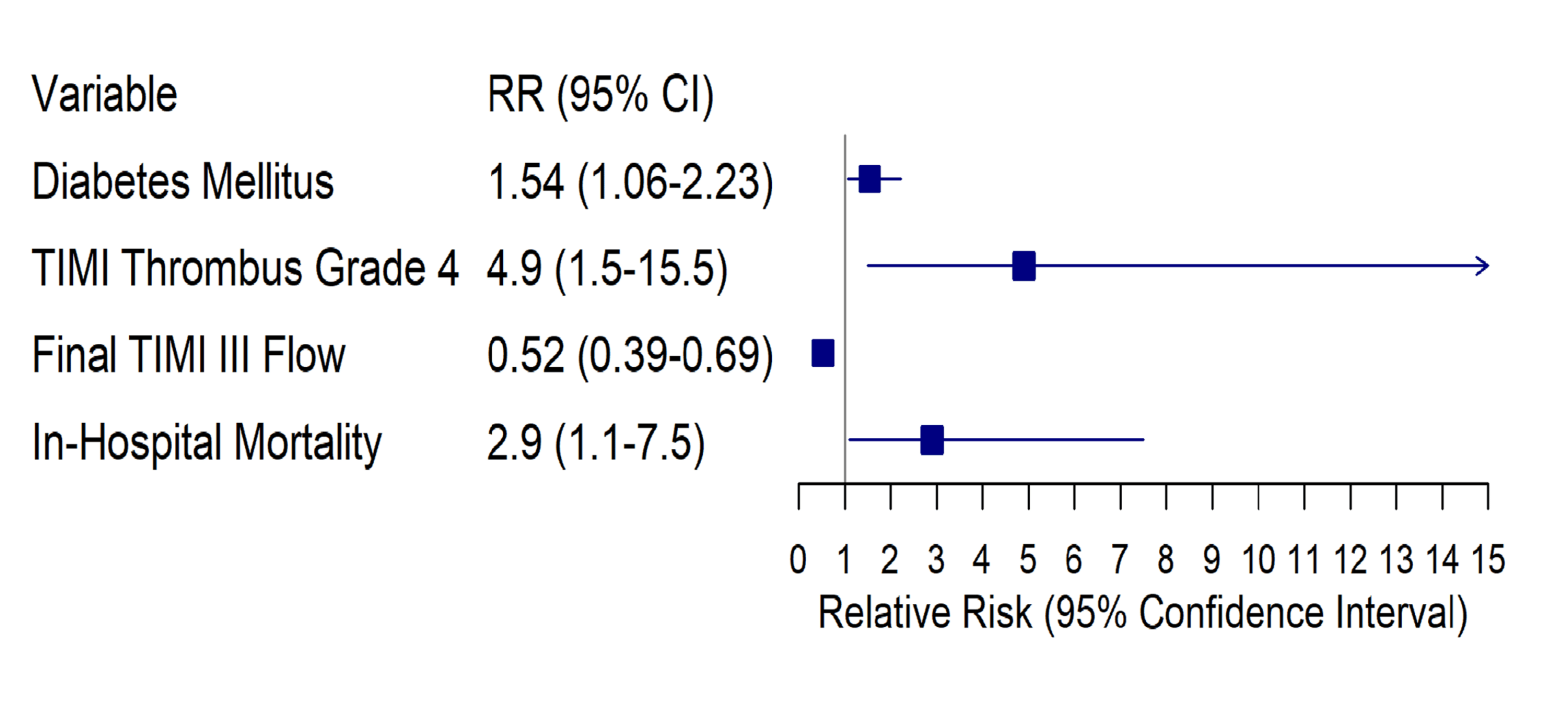
Forest plot of clinical and angiographic correlates of SF/NR Points denote adjusted odds ratios (aORs); horizontal bars, 95% CIs; vertical line, null (OR = 1.0). Estimates correspond to the multivariable model in Table 3 (N = 226; 65 events). Abbreviations: SF/NR: slow flow/no-reflow; TIMI: thrombolysis in myocardial infarction; RR: risk ratio

**Figure 2:**
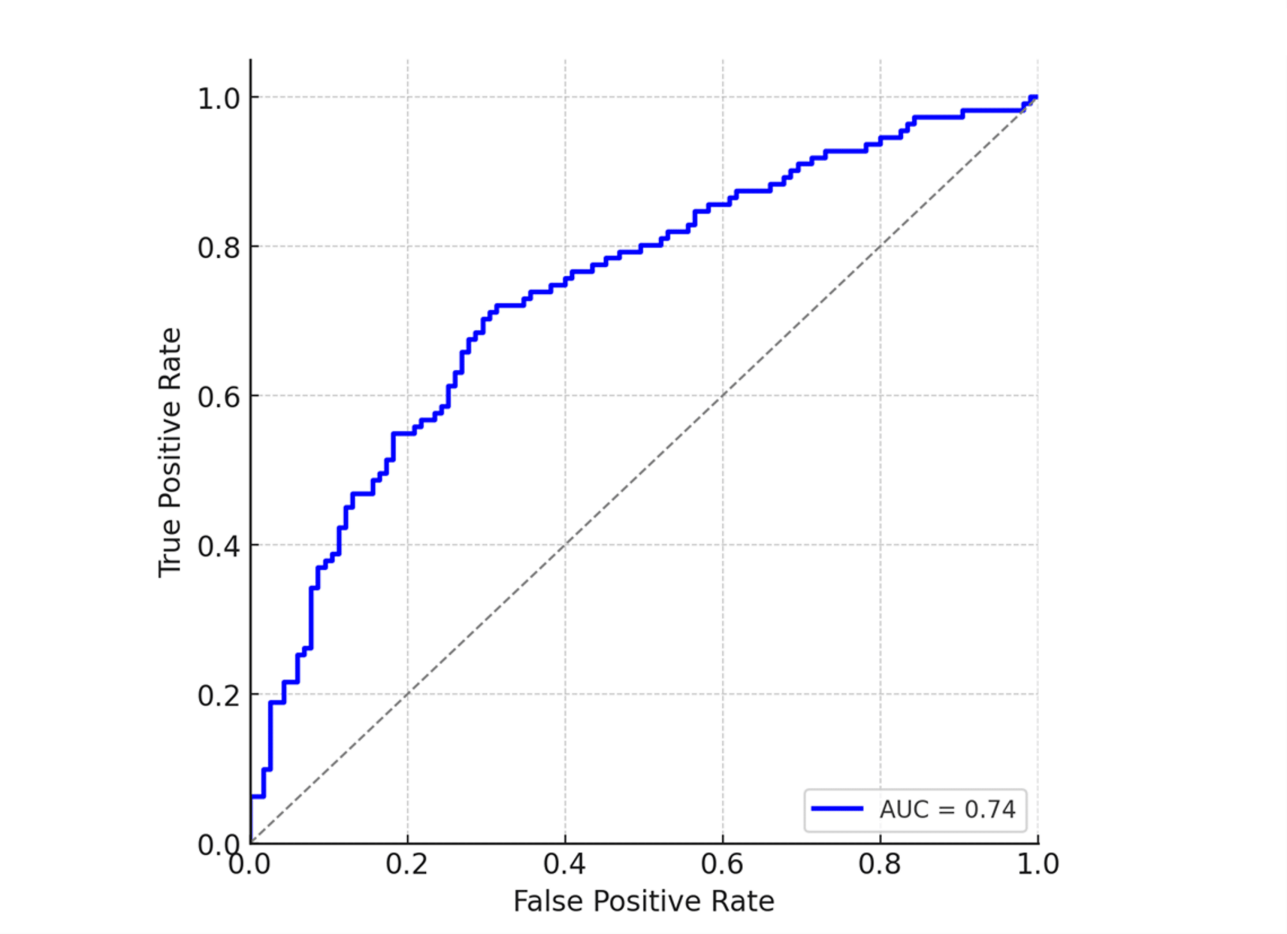
The model demonstrated moderate discrimination (AUC 0.74), with minimal optimism on bootstrap validation (optimism-corrected AUC 0.73). Receiver operating characteristic (ROC) curve for the final model. Abbreviations: AUC: area under the ROC curve

In multivariable logistic regression summarized in Table 3, diabetes mellitus (odds ratio (OR) 2.32, 95% CI 1.05-5.11; p=0.038), higher angiographic thrombus burden (OR 1.93, 95% CI 1.34-2.77; p=0.001), and longer symptom-onset-to-balloon time (per hour increase; OR 1.18, 95% CI 1.04-1.35; p=0.010) remained independently associated with SF/NR. The corresponding adjusted ORs (aORs) and 95% CIs are displayed in Figure 1. The model demonstrated good calibration and moderate discrimination, with an AUC of 0.74 (95% CI 0.65-0.83; p<0.001), as illustrated in Figure 2. 

**Table 3:** Table 3: Multivariable logistic regression predicting SF/NR An asterisk indicates statistical significance (P < 0.05). Multivariable logistic regression model predicting SF/NR after primary PCI. Values are adjusted odds ratios (aORs) with 95% CIs. Candidate covariates were entered based on clinical relevance and/or univariable screening (P < 0.10). Model diagnostics: calibration by Hosmer–Lemeshow test; discrimination by AUC (ROC); internal validation with bootstrap optimism correction; multicollinearity assessed by VIFs, targeting VIF < 2; events-per-variable ≥ 10:1. Two-sided α = 0.05. Abbreviations: aOR: adjusted odds ratio; OR: odds ratio; CI: confidence interval; VIF: variance inflation factor; AUC: area under the curve; ROC: receiver operating characteristic; TIMI: thrombolysis in myocardial infarction; PCI: percutaneous coronary intervention; SF/NR: slow flow/no-reflow.

Predictor	Adjusted OR (95% CI)	P-value	VIF
Diabetes mellitus	2.05 (1.07–3.91)	0.030*	1.12
TIMI thrombus grade 5	3.02 (1.55–5.87)	0.001*	1.09
Pre-PCI TIMI 0 flow	3.85 (1.53–9.67)	0.004*	1.15
Ischemia time (per hour)	1.04 (0.96–1.13)	0.324	1.08

In-hospital complications

Electrical instability was more common with SF/NR: tachyarrhythmias 10.8% vs 3.7% (P = 0.046). Rates of cardiogenic shock, heart failure, bradyarrhythmia, and acute kidney injury were similar between groups. The composite “any complication” occurred numerically more often with SF/NR (18.5% vs 13.0%; P = 0.304), indicating that the excess early risk was driven chiefly by electrical rather than hemodynamic or renal complications, as shown in Table 4.

**Table 4:** In-hospital complications by SF/NR status An asterisk indicates statistical significance (P < 0.05). In-hospital complications stratified by SF/NR status. Values are n (%); P-values from Fisher’s exact test. “Any complication” is a composite defined as ≥1 event from the electrical (tachyarrhythmias, bradyarrhythmias), hemodynamic (cardiogenic shock, heart failure), or renal (AKI) domains. Interpretation: emphasize domain-specific patterns and absolute risks alongside P-values. Available case analysis with cell-level denominators shows if any category has missingness. Abbreviations: SF/NR: slow flow/no-reflow; AKI: acute kidney injury

Domain	Complication	No SF/NR (n = 161)	SF/NR (n = 65)	P-value
Electrical	Tachyarrhythmias	6 (3.7%)	7 (10.8%)	0.046*
	Bradyarrhythmias	3 (1.9%)	2 (3.1%)	0.627
Hemodynamic	Cardiogenic shock	11 (6.8%)	4 (6.2%)	1.000
	Heart failure	6 (3.7%)	4 (6.2%)	0.479
Renal	Acute kidney injury	1 (0.6%)	0 (0.0%)	0.179
Composite	Any complication	21 (13.0%)	12 (18.5%)	0.304

## Discussion

In this prospective, real-world cohort of STEMI patients undergoing primary PCI at a high-volume South Asian center, nearly one-third developed SF/NR. Although reported rates vary with case definitions and adjudication methods, our incidence lies toward the upper end of contemporary series (≈2%-11% in large datasets and >20% in angiography/blush-based cohorts), underscoring a persistent procedural challenge where prolonged ischemia and heavy thrombus burden are common [22-24]. Mechanistically, our findings are consistent with the central roles of distal atherothrombotic embolization, endothelial injury, platelet-leukocyte plugging, vasomotor dysfunction, and reperfusion injury that together produce microvascular obstruction despite restored epicardial patency [1-4,25].

The strongest correlates of SF/NR in our multivariable model, diabetes mellitus, TIMI thrombus grade 5, and pre-PCI TIMI 0 flow, are biologically and clinically plausible and align with prior literature linking metabolic milieu, embolic substrate, and absent antegrade flow to microvascular failure [2-4,22-25]. Contemporary trial data further inform device decision-making in high-thrombus settings [26]. In contrast, total ischemia time did not independently predict SF/NR after adjustment, although longer delays are broadly associated with impaired microvascular integrity in external cohorts and remain an important system-level target [27]. In our cohort, symptom-to-balloon times were uniformly prolonged with relatively limited dispersion between groups, which likely attenuated the ability to detect a more graded, independent time-dependent effect on SF/NR despite the established biological importance of ischemic delay. Practically, a pre-PCI risk triad (diabetes, heavy thrombus, TIMI 0 flow) may help operators anticipate SF/NR before device escalation and consider early microvascular-protection strategies while remaining consistent with guideline-directed care [6,7,24] and should be regarded as a pragmatic risk-flagging concept that requires external validation rather than a formal risk score or causal framework.

Pharmacologic rescue. In our series, most often intracoronary nitrates and a GP IIb/IIIa inhibitor, pharmacologic rescue rarely restored optimal epicardial flow once SF/NR had been declared, mirroring syntheses showing that no single agent reliably reverses established no-reflow across settings [5-7,28,29]. Small comparative studies and meta-analyses suggest potential benefits with non-dihydropyridine calcium-channel blockers (e.g., verapamil/diltiazem) or nicorandil and selective use of adenosine in prevention/treatment, but effect sizes are heterogeneous and context-dependent [28,29]. Distal drug delivery via microcatheter may enhance local bioavailability; feasibility signals exist, although definitive superiority over guide-catheter delivery has not been established [9]. Accordingly, guideline-anchored practice emphasizes prevention, early recognition, and stepwise algorithms tailored to thrombotic burden and vessel size rather than reliance on any single “rescue” drug [5-7]. Our findings regarding pharmacologic use and response are descriptive and should be interpreted within the limits of this observational design.

Device strategy

In high-thrombus lesions, strategy remains nuanced. Routine manual thrombectomy is not supported and may increase stroke risk; selective bail-out use in extreme thrombus remains reasonable but unproven for hard outcomes [26,27]. Within this evidence landscape, careful catheter manipulation, staged lesion preparation, judicious glycoprotein IIb/IIIa use in selected cases, and consideration of embolic-protection tools in appropriate anatomies represent pragmatic levers that can be protocolized within resource-constrained systems [6,7]. These device-related observations in our cohort are hypothesis-generating and are intended to complement, rather than supersede, randomized trial evidence and guideline recommendations.

Our data also reinforce the clinical salience of SF/NR; affected patients had substantially lower final TIMI 3 flow and more early adverse events, particularly ventricular arrhythmias, consistent with broader evidence linking microvascular obstruction to larger infarcts, reduced myocardial salvage, electrical instability, and adverse outcomes [3,4,27,30]. While we did not collect long-term endpoints, the literature indicates that no-reflow carries durable prognostic implications beyond discharge, underscoring the need for upstream risk mitigation and intra-procedural vigilance [3,4,27,30]. Taken together, the associations observed in this study should be viewed as supporting evidence for risk stratification and quality improvement in primary PCI rather than as proof of causality.

Limitations

This observational, single-center analysis may limit generalizability; however, it reflects an unselected, real-world STEMI population with consecutive enrollment and standardized data capture consistent with reporting guidance [11]. The sample size constrained precision for some subgroup estimates; to limit overfitting, we prespecified covariates, assessed collinearity, evaluated model calibration, and maintained events-per-variable ratios in line with methodological recommendations [18-21]. In addition, ischemia times in this cohort were generally prolonged with relatively little variability between groups, which may have limited our ability to explore more subtle, graded associations between incremental ischemic delay and the risk of SF/NR or in-hospital outcomes. To avoid unstable, underpowered subgroup estimates, we did not further subdivide ischemia time into narrow hourly categories; the absence of an independent adjusted association in our model should therefore be interpreted in the context of these design and distributional constraints rather than as evidence against the biological relevance of ischemic delay. Long-term outcomes were not captured; nonetheless, short-term clinical associations (e.g., ventricular arrhythmias) were prospectively recorded and align with external evidence on the prognostic relevance of SF/NR, supporting biological plausibility [3,4,27,30]. Therapeutic choices (e.g., thrombectomy and intracoronary pharmacotherapy) were at the operator's discretion, introducing confounding by indication; even so, practice patterns broadly reflected contemporary guidance and major trials [6,7,26,27]. Quantitative microvascular metrics (e.g., index of microcirculatory resistance) and systematic details on distal drug delivery were unavailable; future work incorporating standardized microcirculatory assessments and device-level granularity is warranted. Finally, we used a pragmatic, angiography-based definition of SF/NR rather than cardiac MRI; while this may underestimate microvascular obstruction, it mirrors routine catheterization-laboratory practice and enhances bedside applicability. Overall, given the observational nature of the study, all reported relationships should be interpreted as associations rather than causal effects.

## Conclusions

In contemporary primary PCI for STEMI, SF/NR was common and most strongly associated with heavy thrombus, absent antegrade flow, and diabetes. Once present, pharmacologic rescue seldom normalized flow, and early complications, especially electrical instability, were more frequent. A simple pre-PCI risk triad may help flag higher-risk cases and prompt preventive, stepwise microvascular-protection strategies aligned with current guidance. Multicenter validation of streamlined prevention-and-rescue protocols is warranted.
